# Sex practices and awareness of Ebola virus disease among male survivors and their partners in Guinea

**DOI:** 10.1136/bmjgh-2017-000412

**Published:** 2017-09-25

**Authors:** Mandy Kader Kondé, Moustapha Keita Diop, Marie Yvonne Curtis, Abdoulaye Barry, Saidou Kouyaté, Ludovica Ghilardi, Sékou Kouyaté, Aissatou Malal Diallo, N’faly Magassouba, Isadora Quick, Mory Keïta, Miles W Carroll, Josep Jansa, Lorenzo Subissi

**Affiliations:** 1 Department of Public Health, Universite Gamal Abdel Nasser de Conakry, Conakry, Guinea; 2 Fondation Santé et Développement durable, FOSAD-CEFORPAG, Conakry, Guinea; 3 Laboratoire d’analyse socio-anthropologique de Guinée (LASAG), Université Général Lansana Conté de Sonfonia-Conakry, Conakry, Guinea; 4 Department of Disease Control, Faculty of Infectious and Tropical Disease, London School of Hygiene and Tropical Medicine, London, UK; 5 Laboratoire des Fièvres Hémorragiques en Guinée, Université Gamal Abdel Nasser de Conakry, Conakry, Guinea; 6 Institut thématique multi-organismes I3M (Immunologie, Inflammation,Infectiologie et microbiologie), Institut National de la Santé et de la Recherche Médicale, Paris, France; 7 WHO Country office, World Health Organization, Conakry, Guinea; 8 Microbiology services, Public Health England, London, London, UK; 9 University of Southampton, South General Hospital, Southampton, UK; 10 Section of Epidemic Intelligence and Response, European Centre for Disease Prevention and Control, Stockholm, Stockholms Län, Sweden; 11 Global Outbreak Alert and Response Network, World Health Organization, Geneva, Switzerland

**Keywords:** epidemiology, health education and promotion, public health, screening, viral haemorrhagic fevers

## Abstract

**Introduction:**

Towards the end of the 2013–2016 West African outbreak, sexually-transmitted Ebola virus re-emerged from Ebola virus disease (EVD) survivors in all three hardest hit countries. We explore sex practices and awareness of the risk of Ebola virus transmission among EVD survivors and their partners.

**Methods:**

In this cross-sectional study, we recruited a convenience sample of study participants aged >15 years who were male EVD survivors, their sexual partners and a comparison group. We administered a questionnaire to all respondents, estimated self-reported sexual practices and risk awareness and conducted in-depth interviews.

**Results:**

We recruited 234 EVD survivors, 256 sexual partners of survivors and 65 individuals in the comparison group from five prefectures in Guinea. The prevalence of safe sexual behaviour (regular condom use or sexual abstinence >12 months) and regular condom use in EVD survivors was 38% (95% CI 31% to 44%) and 21% (95% CI 16% to 27%), respectively. Among partners, these prevalences were lower (11%, 95% CI 7% to 15% and 9%, 95% CI 5% to 12%, respectively). EVD survivors were more than five times as likely to engage in safe sexual behaviour compared with the comparison group (aOR 5.59, 95% CI 2.36 to 13.2). One-hundred and thirty one EVD survivors (57%) and 94 partners (37%) were aware of the risk of Ebola virus re-emergence associated with having unsafe sex. Partners who reported not being informed by their husband/boyfriend (EVD survivor) were more likely to be unaware of this risk (aOR 20.5, 95% CI 8.92 to 47.4).

**Conclusions:**

We disclose here a need to improve knowledge of the disease and close the gap between knowledge and practice found in EVD survivors and their partners. Current and future survivors’ follow-up programmes should include partners and be more effective at communicating sex-related risks. Community-level fears and attitudes that enable stigmatisation should be addressed. Safe sex interventions targeting EVD survivors and their partners should be prioritised.

**Key questions**

**What is already known about this topic?**

- One study from Liberia reported EVD survivor sexual behavioural data, but had no comparison group and was mainly focused on semen testing.
- One study from Sierra Leone reported qualitative findings from in-depth interviews with survivors, including their knowledge on sexual transmission risk.
- None of the previous studies explored awareness of the risk of sexual transmission among EVD survivors and/or partners.

**What are the new findings?**

- Ebola survivors were five times as likely to engage in safe sex practices, relative to the comparison group.
- A low prevalence of reported safe sex practices among survivors and their partners.
- More than half of the partners were unaware of the risk of getting Ebola associated with having unsafe sex with a survivor, and most often the only source of information was the survivor himself.

**Recommendations for policy**

- All EVD survivors’ follow-up programmes should also enrol their sexual partners.
- International guidelines on communication of the risk of EVD sexual transmission should be better implemented to address gaps in knowledge, risk perceptions and safe sex practices, diffused in this study even among EVD survivors.
- This study will help to shape survivors’ follow-up programmes in West Africa and wherever Ebola may emerge next.

## Introduction

Since the discovery of the Ebola virus in northern Democratic Republic of Congo (at that time Zaire) in 1976, sporadic Ebola virus disease (EVD) outbreaks have been regularly reported in central Africa.^1^ In 2013–2016, an EVD outbreak severely hit West Africa (mostly Guinea, Liberia and Sierra Leone) and infected, over 2  years, more than 28 616 people, of whom around 11 310 died.^2^ The West African cohort of EVD survivors—the largest in history—is shedding new light on the disease, from a range of post-Ebola sequelae to viral persistence in immune-privileged sites.^3–7^ Because infectious Ebola virus can persist for more than 15 months in the semen,^8 9^ it can cause EVD re-emergence. After the original outbreak faded, at least 10 such episodes (ie, new infections most likely attributable to contact with infectious semen of EVD survivors) happened in all three hardest hit countries.^9–13^ The World Health Organization (WHO) interim advice on this topic recommends safe sexual practices for 12 months after symptom onset or until the survivor’s semen tests negative for Ebola virus twice.^14^ A modelling study based on 26 EVD survivors from Guinea predicted that, by July 2016, all West African EVD survivors should have experienced viral clearance in the semen.^15^ Viral persistence was found to be associated with age, younger men (<40 years) being more likely to test negative for Ebola virus than older ones.^8^ The same study also points at a positive impact of sexual health promotion on survivors’ safe sex practices.^8^ A qualitative study from Sierra Leone reported that EVD survivors’ knowledge about sexual transmission risk reflected counselling messages.^16^ However, those interventions did not include sexual partners of EVD survivors, and their level of awareness of risk of Ebola virus transmission is unknown.

Communication to EVD survivors of the viral persistence in their semen has been difficult in Guinea. Evidence that infectious virus could be hosted in the semen for longer periods of time has steadily grown throughout the outbreak. Therefore, research findings could not be easily translated in strict recommendations.^6 14 17^ Ebola Treatment Unit (ETU) doctors, often from NGOs like Médecins Sans Frontières, communicated the latest discoveries to EVD survivors at their release, which translated in recommendations to practice sexual abstinence or consistent condom use. First, the recommendation was for 3 months, then for 6 months, and finally 12 months or after two RT-PCR-negative semen samples. By the end of the outbreak, research programmes were coordinated by the National Coordination for the fight against Ebola (CNLEB) to ensure a nationwide semen testing programme which also informed EVD survivors on sexual health issues.^7 15^

To better understand the risk of EVD re-emergence due to sexual transmission after the end of an EVD outbreak, it is critical to understand the barriers that may be encountered in communicating with patients who survived EVD and have to deal with the stigma associated with it.^18^ We report here results from a cross-sectional study aimed at comparing sex practices and awareness of the risk of EVD sexual transmission among survivors and their sexual partners (hereafter partners).

## Methods

### Study design

EVD male survivors were eligible if aged >15 years and able to show the certificate that every survivor was given at discharge from the ETU, which confirmed their Ebola-free status in the blood. They were recruited using databases from the CNLEB, WHO and USA Centres for Disease Control (CDC), which in total had records of 491 EVD male survivors aged >15 years. These lists were compared with those of the EVD survivors’ associations in each local community. We aimed to recruit as many eligible survivors as possible. Because many were lost to follow-up (ie, moved to another community, had fake number or no contact), we only invited 240 (49%) of the eligible survivors (Supplementary figure). Invited EVD survivors were asked to bring along their sexual partner(s). For every three recruited EVD survivors, we aimed to recruit one individual from the same neighbourhood for the comparison group. For this group, we managed to recruit only 83% (65/78) of the original sample size because of the stigma around the disease (many EVD survivors did not talk about the disease to their relatives or did not want them to know about their disease). The comparison group was recruited with the help of the Ebola survivors’ associations using random digit dialling from a list of up to 20 relatives or acquaintances given by each survivor at enrolment. Individuals in the comparison group were from the same communities as survivors and partners. They were not matched to them, but each regression model we adjusted for region and zone of residence. Participants were recruited in the prefectures of Coyah and Forécariah (lower Guinea), Macenta and Guéckédou (forested Guinea), and in the capital, Conakry. Recruitment of study participants started on 21 April 2016 in Forécariah and ended on 23 June 2016 in Guéckédou. All interviews were face-to-face and were done at the prefectural centres of the CNLEB in urban areas, and in the closest healthcare centres in rural areas. They were always done in private rooms, in the presence of only the interviewer and the interviewee, and the latter could choose the preferred language for the interview between French and one of the local languages (Susu, Malinké, Pular and Konianké). All interviewers were either medical doctors or socioanthropologists from Guinea, able to speak one or more local languages in addition to French, and trained in Good Clinical Practice. Every participant signed a written informed consent. The following data collection tools were used: three questionnaires, one for each group (ie, survivors, partners and the comparison group), a concept paper for HIV/syphilis counselling and a document to report the HIV or syphilis-positive cases. In addition, recording devices were used for in-depth interviews.

10.1136/bmjgh-2017-000412.supp1 Export to PDFSupplementary file 1

Sexual behaviour, EVD awareness and other indicators were assessed through a questionnaire. After the interview, a counselling session was done to explain the use of a HIV/syphilis dual Rapid Diagnostic Test (RDT) and the consequences of a potential positive diagnosis, following the guidelines of the National Committee for the fight against AIDS (CNLS). The RDT was administered to all consenting EVD survivors and a sexual health promotion intervention strictly recalling WHO interim advice on EVD sexual transmission was given to everybody.^14^ Long-acting benzathine penicillin G for single intramuscular injection was prescribed to syphilis-positive EVD survivors. HIV- and syphilis-positive patients were referred to the closest hospital to access treatment.

### Questionnaire

The questionnaire was interviewer-assisted. The part of the questionnaire on sex practices was completely based on UNAIDS questions to assess sexual behaviour.^19^ Safe sexual behaviour was defined as reporting regular condom use (using condom at each sexual intercourse) or sexual abstinence (>12 months). Only for EVD survivors, the following UNAIDS indicators were collected: (i) higher risk sex in last year (‘higher risk sex’ refers to sex with a casual partner compared with sex with primary sexual partner); (ii) condom use at last higher risk sex; and (iii) commercial sex in last year.

Concerning risk awareness of EVD sexual transmission, virologists, clinicians, epidemiologists and socioanthropologists participating in the study, with experience in HIV/AIDS and Ebola research in sub-Saharan Africa, agreed on six questions to assess awareness of risk of EVD sexual transmission with a score from 0 to 6 (Supplementary file). Young individuals who reported not having had first-time sex and old ones who reported having stopped sexual activity were excluded from the awareness analysis. This short questionnaire was validated using a pilot study: the six questions about Ebola virus persistence and the risk of sexual transmission were put to a small random sample of people (n=15) with diverse educational backgrounds, recruited through the Ebola associations on a voluntary basis, to validate the representativeness of the awareness score. These people were not included in the final study. The validation was done comparing the scores with the true knowledge of participants on the subject, in its turn assessed through in-depth interviews with all participants of the pilot study. Based on the result of the pilot study, the awareness score was then transformed into a binary variable (<4, not aware; >4, aware). Zone of residence (urban versus rural), region of residence, age, years in education and employment (this last divided into three categories based on the expected income: (i) lower income: housewife/farmer/labourer; (ii) higher income: State employee/business owner; and (iii) unpredictable income: other) were treated as potential confounders. Zone of residence was assessed by interviewers based on the address of residence declared by the study participants.

10.1136/bmjgh-2017-000412.supp2 Supplementary file 2

### Statistical analysis

We used STATA 13.0 to perform statistical analyses. For binary and categorical variables, we reported the number of observations (n) and the percentage (%), while for continuous variables we reported the median and IQR. For comparison between groups, Pearson χ^2^ test (or Fisher exact test if few observations) was used. For comparison between categories of the same variable, the Wald test was used. To obtain an estimation of the p value of the association of a categorical variable with a binary outcome, the Likelihood ratio test (LRT) was used, which compared the full model with a model without the exposure of interest. We used logistic regression to look at the association of sexual behaviour with study group and examine factors associated with awareness of the risk of sexual transmission of Ebola virus among survivors and their partners. To adjust for the study design, region of residence and zone of residence (urban versus rural) were forced variables of each model. Age, years in education and employment were treated as potential confounders. For logistic regression data reporting, we showed the number of individuals with outcome among the individuals with the exposure of interest (n/N). Interactions of region of residence with zone of residence (urban versus rural) as well as years in education with employment, region of residence and zone of residence were explored by LRT using final logistic regression models with the following outcomes: (i) sexual behaviour and (ii) risk awareness among EVD survivors. Since missing data were few (<2% for all variables used in the main analysis), they were excluded from the analyses.

### Qualitative data collection and analysis

In-depth individual interviews were mainly used to clarify, confirm and follow up on key issues examined in the survey. In total, 25 EVD survivors, 25 partners and 10 individuals of the comparison group were interviewed in the five different prefectures of the study. Participants were chosen using the following criteria: time passed from ETU discharge for survivors (for half <8 months, for the other half >15 months); (i) younger age (18–45, because they were expected to be sexually active and more open to questions about sexual behaviour); and (ii) openness to answer questions about sex during the first questionnaire for all study participants. Thematic analysis was used to define the main clusters of concepts related to the knowledge of the disease, the awareness of risk of transmission of Ebola virus and the barriers to condom use for EVD survivors and partners. Interviews were done in French, Susu, Malinké, Pular and Konianké, depending on the preference of each participant.

In addition, 11 stakeholder interviews with health professionals (n=5) and NGO workers (n=6) were carried out to explore challenges faced when informing about the EVD transmission risk after recovery. Volunteer stakeholders were recruited in the local hospital of the prefectures visited during the study. Interviews were registered on recording devices and transcribed by two independent interviewers before analysis. Direct observations (eg, interactions between interviewer and interviewee) were used to contextualise the individual interviews.

## Results

### Characteristics of the study participants

**Figure 1:**
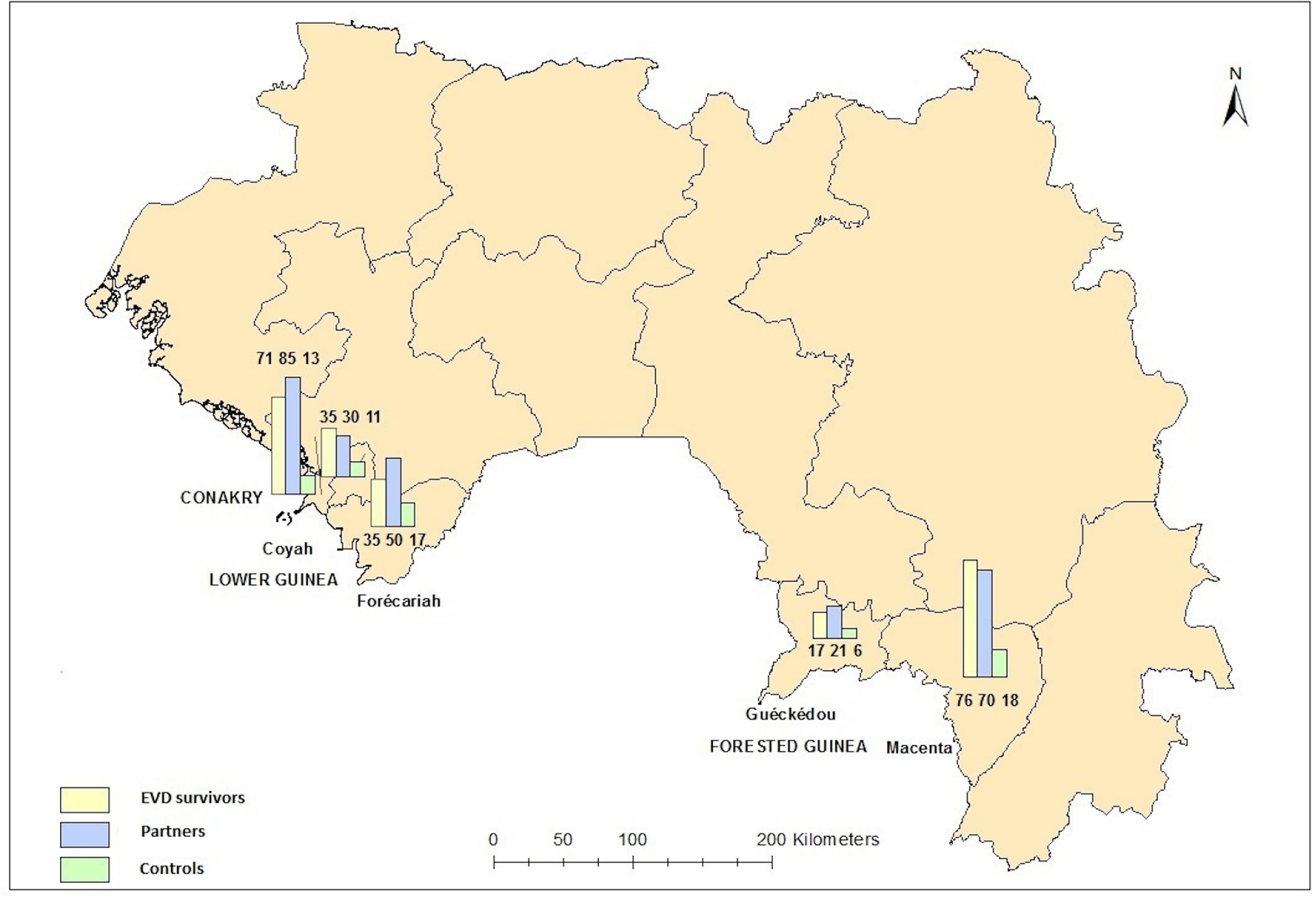
Number of enrolled participants by study group and prefecture.

We invited 240 EVD male survivors to participate in the study. From 21 April to 23 June 2016, 234 of them were recruited in five prefectures (response rate 98%, figure 1). For all EVD survivors, the median duration between discharge from an ETU and study enrolment was 557 days (IQR 440–733). We asked the participants to bring their sexual partner(s) to the interview. Of them, 42 came alone, 136 (58%) came with one partner, 51 (22%) with two partners and 5 (2%) with three partners. All partners were females. Of the 256 recruited partners, 187 were married to their respective EVD survivor (73%). We also recruited 65 individuals, of which six were females (9%), for the comparison group. Nearly a third (30%) of the study participants were from Conakry, 32% from lower Guinea (Coyah and Forécariah) and 38% from forested Guinea (Guéckédou and Macenta), where the epidemic originated in December 2013.^20^ The overall median age was 28 (IQR 22–35). Age was similar between the comparison group (31, IQR 25–38) and survivors (32, IQR 25–41), but partners were younger (25, IQR 20–30, p<0.001, Pearson χ^2 ^test). Survivors were less educated than the comparison group (p=0.003, χ^2 ^test), while there was no difference in employment between the two groups (table 1).

**Table 1:** Sociodemographic characteristics of the study participants

	EVD survivors (n=234)	Partners (n=256)	Comparison group (n=65)	p Value*
	n (%) or median (IQR)	n (%) or median (IQR)	n (%) or median (IQR)	
Age (years)	32 (25–41)	25 (20–30)	31 (25–38)	
15–24	58 (25)	117 (46)	15 (23)	0.037
25–39	110 (47)	123 (48)	35 (54)	
40–59	57 (24)	14 (6)	8 (12)	
>60	9 (4)	0 (0)	7 (11)	
Gender				
Male	234 (100)	–	59 (91)	–
Female	–	256 (100)	6 (9)	
Years in education				
None	82 (35)	143 (56)	37 (35)	0.003
1–5	39 (17)	39 (15)	48 (45)	–
6–12	71 (30)	58 (23)	22 (21)	–
>13	41 (18)	15 (6)	21 (32)	–
Employment				
State employee/business owner	62 (27)	77 (30)	15 (23)	0.60
Housekeeper/farmer/labourer	95 (41)	134 (53)	24 (38)	–
Other	75 (32)	44 (17)	25 (39)	–
Religion				
Muslim	171 (74)	200 (78)	49 (75)	0.96
Christian	57 (25)	51 (20)	15 (23)	
Other	4 (2)	4 (2)	1 (2)	
Prefecture				
Conakry	71 (30)	85 (33)	13 (20)	0.17
Coyah (lower Guinea)	35 (15)	30 (12)	11 (17)	–
Forécariah (lower Guinea)	35 (15)	50 (20)	17 (26)	–
Guéckédou (forested Guinea)	17 (7)	21 (8)	6 (9)	–
Macenta (forested Guinea)	76 (32)	70 (27)	18 (28)	–
Zone of residence				
Urban	120 (51)	130 (51)	30 (46)	0.46
Rural	114 (49)	126 (49)	34 (54)	

*Pearson χ^2^ test compares survivors with the comparison group, excluding partners.

### Sexual behaviour

In total, 145 (26%) participants reported safe sexual behaviour (38% of EVD survivors, 11% of partners and 14% of the comparison group). Of those, 81 reported regular condom use (22% of survivors, 9% of partners and 14% of the comparison group), while 42 reported sexual abstinence (15% of survivors, 2% of partners and none of the comparison group) (table 2). The inconsistency between sexual behaviour in survivors and partners is, at least to some extent, due to second and third partners (22% of all recruited partners) of the same EVD survivor.

**Table 2:** Sexual behaviour and awareness of risk of Ebola sexual transmission among participants and prevalence of UNAIDS indicators among Ebola survivors

	EVD survivors (n=234) n (%)	Partners (n=256) n (%)	Comparison group (n=65) n (%)	p Value*
Safe sexual behaviour				
Yes	87 (38)	27 (11)	9 (14)	<0.001
No	145 (62)	226 (89)	55 (86)	–
Sexual abstinence				
Yes	37 (16)	5 (2)	0 (0)	<0.001
Regular condom use				
Yes	50 (22)	22 (9)	9 (14)	<0.001
Aware of risk				
Yes (score >4)	131 (57)	94 (37)	30 (47)	<0.001
No (score <4)	100 (43)	160 (63)	34 (53)	–
Score, median (IQR)	4 (0–5)	3 (1–4)	3 (2–5)	–
Prevalence of UNAIDS indicators (%)				
Higher* risk sex in last year	37	–	–	
Condom use at last higher* risk sex	27	–	–	
Commercial sex in last year	5	–	–	

Safe sexual behaviour was defined as regular condom use (ie, always using condoms at sexual intercourse or sexual abstinence for at least 12 months).

*Higher compared with sex with primary sexual partner.

Among EVD survivors, some UNAIDS sexual behaviour indicators were also estimated^21^: 37% of them had had higher risk sex in the last year (higher compared with sex with primary partner—that is, sex with a casual partner) and, amongst them, 27% reported having used a condom at last higher risk sex. Of those who last had unsafe higher risk sex, 9 (10%) had been discharged for less than a year, 47 (51%) for 1–1.5 years, 28 (30%) for 1.5–2 years and 8 (9%) for >2 years. 5% (12/233) of EVD survivors reported having had sex with a commercial sex worker (CSW) in the last year (table 2), of which one reported no condom use. Of those, three had been discharged from the ETU for more than 2 years, three for 1.5–2 years, five for 1–1.5 years and one for less than a year.

The prevalence of other sexually transmitted infections (STIs) among EVD survivors was low (1% for HIV type 1 and 1% for syphilis, none was HIV type 2-positive). Of the four STI-positive subjects (two for HIV type 1, two for syphilis), none reported sex with a CSW in the last year. All four reported having had unsafe sexual behaviour and no use of condom the last time they had sex with a casual partner.

Unmarried participants were more than twice as likely to have safe sexual behaviour than married ones (p=0.001, Wald test). EVD survivors were five times as likely to have safe sexual behaviour (aOR 5.59, 95% CI 2.36 to 13.2) than the comparison group (table 3). However, partners did not have safer sexual behaviour than the comparison group (p=0.73, Wald test). The difference in the behaviour of survivors and that of the comparison group was confirmed when regular condom use (instead of safe sexual behaviour, therefore excluding sexual abstinence) was used as the outcome (aOR 2.48, 95% CI 1.02 to 5.99, table 3). Participants from Conakry were more than three times as likely to use a condom regularly than those from forested Guinea (p=0.007, Wald test).

**Table 3:** Association of sexual behaviour (safe versus unsafe sex) and regular condom use with study group (survivor, partner and comparison group)

	Sexual behaviour			Regular condom use		
	n/N (%)	OR (95% CI)	aOR* (95% CI)	n/N (%)	OR (95% CI)	aOR* (95% CI)
All	123/549 (22)			81/549 (15)		
Study group						
Comparison group	9/64 (14)	–	–	9/64 (14)	–	–
EVD survivors	87/232 (38)	3.67 (1.73 to 7.79)	5.59 (2.36 to 13.2)	50/232 (22)	1.68 (0.78 to 3.63)	2.48 (1.02 to 5.99)
Partners	27/253 (11)	0.73 (0.32 to 1.64)	0.68 (0.05 to 5.92)	22/253 (9)	0.58 (0.25 to 1.33)	0.46 (0.04 to 5.21)

*Adjusted for age, marital status, zone of residence (urban versus rural), years of education, gender and region of residence (divided into Conakry, lower Guinea and forested Guinea).

n, participants with outcome (safe sexual behaviour); N, total number of exposed participants; OR, odds ratio; aOR, adjusted OR.

Safe sex practices included (1) regular condom use, defined as always using condoms at sexual intercourse, and (2) sexual abstinence for at least 12 months. The comparison group served as the reference group.

### Awareness of risk of sexual transmission of Ebola virus

In total, 255 participants (46%) including 131 survivors (57%), 94 partners (37%) and 30 individuals in the comparison group (47%) were aware of the risk of EVD sexual transmission (table 2). Among those, 187 (75%) were aware that the virus can persist for 9 months or more in the semen, while 19% (n=48, of which 27 were survivors) thought the virus could not persist for more than a month. Those survivors were not those discharged from the ETU earlier in the outbreak (p=0.58), when the persistence of Ebola virus was not known potentially to last so long.

EVD survivors were almost three times as likely to be aware of the risk of EVD transmission after recovery than the comparison group (aOR 2.85, 95% CI 1.45 to 5.59, table 4). Among partners, 115 (51%) reported having been informed by their husband/boyfriend about the risks of EVD sexual transmission (Supplementary table 1). This was strongly associated with their awareness of EVD transmission potential after recovery (aOR 20.5, 95% CI 8.92 to 47.4). Age was associated with risk awareness among partners, middle-aged women (aged 25–39 years) being almost three times as likely to be aware of the risk compared with younger women (aged 15–24 years, p=0.027, LRT), but not among EVD survivors (p=0.23, LRT). EVD survivors who had >13 years of education were 10 times as likely to be aware of the disease transmission potential after recovery than those with no education (p<0.001, LRT). Similarly, those who were state employees or business owners were almost three times as likely to be aware of the risk compared with the lower-income work category consisting of labourers, farmers and housekeepers (p<0.001, LRT). EVD survivors from outside Conakry (from both lower or forested Guinea) were roughly 3–4 times as likely to be aware of the risk compared with those from Conakry (p=0.009, LRT, Supplementary tables 1 and 2).

**Table 4:** Association of awareness of risk of Ebola virus sexual transmission and study group

	n/N (%)	Crude OR (95% CI)	aOR* (95% CI)
All	161/299 (53.8)		
Study group			
Comparison group	30/65 (46.1)	–	–
Survivors	131/234 (56.0)	1.48 (0.85 to 2.58)	2.85 (1.45 to 5.59)

Partners, all women, were excluded from this analysis because too few individuals in the comparison group were females.

*Adjusted for age, employment, years of education, region of residence and zone of residence.

### Qualitative findings

We report here key findings that emerged from the qualitative analysis to help contextualise survey results: in-depth interviews confirmed that EVD survivors are reluctant to talk about viral persistence in their semen. Some EVD survivors had lost a wife/girlfriend from EVD and did not want to inform their new partner as they feared stigma and/or losing her. EVD survivors already experienced physical and emotional pain as well as deep stigmatisation. They perceived informing the partner about their potential infectiousness as being sick. Some were even forced to move to another community:

 "Within our community of origin, no head of the family would accept to give us his daughter in marriage, because they all associate us to Ebola".

Moreover, increasing scientific knowledge on the potential length of viral persistence led to contrasting recommendations: the length of the suggested period of sexual abstinence increased throughout the outbreak at release from ETU and during follow-up programmes. This created confusion among EVD survivors and some lost their faith in health authorities.

In-depth interviews also showed that the vast majority of the study participants associated condom use with infidelity. Other main barriers were lack of sexual pleasure, conception desire and lack of knowledge. The latter includes popular beliefs such as condom use leading to infection or condom possibly ‘exploding’ during sexual intercourse. Few participants reported refusing condom use because it was unacceptable for their faith.

Stakeholder interviews with health workers from public and NGO structures highlighted the challenge for health workers to communicate to a cured person the potentially long-lasting persistence of the virus.

## Discussion

We report here a low prevalence of safe sexual behaviour and disease awareness among EVD survivors and their partners. This study highlights a need to improve communication on the persistence of the Ebola virus, which should include recruitment of partners of EVD survivors in follow-up programmes.

The prevalence of condom use, which was higher in EVD survivors than in the comparison group, was comparable in 113 Liberian EVD survivors (26% vs 21% in this study).^8^ HIV prevalence among EVD survivors was 1%, in line with the estimate of 1.3% in the general population.^22^

As expected, EVD survivors also had a significantly higher awareness of risk of Ebola virus sexual transmission than the comparison group. Survivors from the capital, Conakry, were less likely to be aware of this risk than those from lower Guinea and forested Guinea. These results are difficult to interpret and may be unreliable due to a potential interaction between region of residence and years in education that could not be fully explored due to lack of data (Supplementary table 1).

Within the partners’ group we found very strong evidence for an association of low risk awareness with being informed by their husband/boyfriend (EVD survivor), which clearly shows that the source of information for most of the partners was exclusively their husband/boyfriend. As for the HIV/AIDS epidemic, young women (15–24 years) are the most vulnerable because of their limited access to information and high dependency on the partner.^23 24^

Our results are comparable to those of another similar study in Liberia, and may be applicable to other Ebola-affected populations in the sub-Saharan African region.^8^

We believe that sexual health promotion interventions, including to partners of EVD survivors, should be inserted in the national semen testing and counselling programme in all hardest hit countries and in any other country that will be affected by EVD in the future. In a future outbreak, sexual partners should be present at ETU release of their husband/boyfriend so that the first intervention can be made before high-risk sexual intercourse may occur; counselling efforts to identify all sexual partners, including casual partners and those from polygamous marriages, should be fostered.

The recent emergence of Ebola virus in West Africa and the consequent recent discovery of its long persistence in the semen should raise awareness of the importance of prioritising behavioural, social support and psychosocial interventions, focusing not only on EVD survivors but also on their sexual partners, which include casual partners and those partners in polygamous marriages, common in the three Ebola-affected countries. In fact, this lack of EVD knowledge among at-risk couples and, in particular, among partners is alarming and must be urgently addressed. Additional counselling on disclosure among partners, as in the case of AIDS, needs to be developed.

Health and education professionals and survivors’ associations need to keep working at the community level against the stigma associated with EVD survivors. EVD survivors’ associations could potentially play an important role in awareness and sensitisation. However, it is important that associations are not manipulated by personal or financial reasons. To avoid this, they could be integrated in the national public health system.

Future research should focus on improving clinical and virological knowledge about the duration of virus persistence in semen, important to improve awareness among health workers and, as a consequence, among the general population.

Our study is mainly based on self-reporting information, and it may therefore be susceptible to social desirability bias, in particular for the group of EVD survivors, leading to an overestimation of safe sexual behaviour. However, the prevalence of safe sexual behaviour was relatively low even among EVD survivors, and comparable to other studies.^8^ Moreover, the information from partners, less prone to social desirability bias, highlighted the lack of sexual health measures taken by many at-risk couples.

Selection bias may have occurred among the individuals recruited in the comparison group. Although they were recruited by random digit dialling by the heads of local survivors’ associations from a list of contacts (relatives and acquaintances) of the EVD survivor, the response rate is unknown and most of the individuals were males and highly educated. The lack of female individuals in this group was not planned, and it was likely due to the higher reluctance of Guinean women, compared with men, to talk about sexual practices. Therefore, the comparison group was mainly used to draw conclusions when compared with male EVD survivors rather than female partners.

## Conclusion

Ebola re-emergence from an EVD survivor has not been reported for over a year. Future efforts should focus on avoiding low-risk awareness coupled with risky sex practices and Ebola-related stigma that may influence disclosure following an Ebola outbreak, especially after widespread transmission that produces large cohorts of male survivors. We recommend that sexual health interventions, including counselling services in the presence of sexual partners, become an integral component of Survivor Care Packages post-discharge to minimise the possibility of sexual transmission due to viral persistence.
