# Supplementary figures and images for: Sex practices and awareness of Ebola virus disease among male survivors and their partners in Guinea
Source: BMJ Glob Health. 2017 Sep 25;2(3):e000412. doi: 10.1136/bmjgh-2017-000412 (PMC5623339; doi:10.1136/bmjgh-2017-000412)

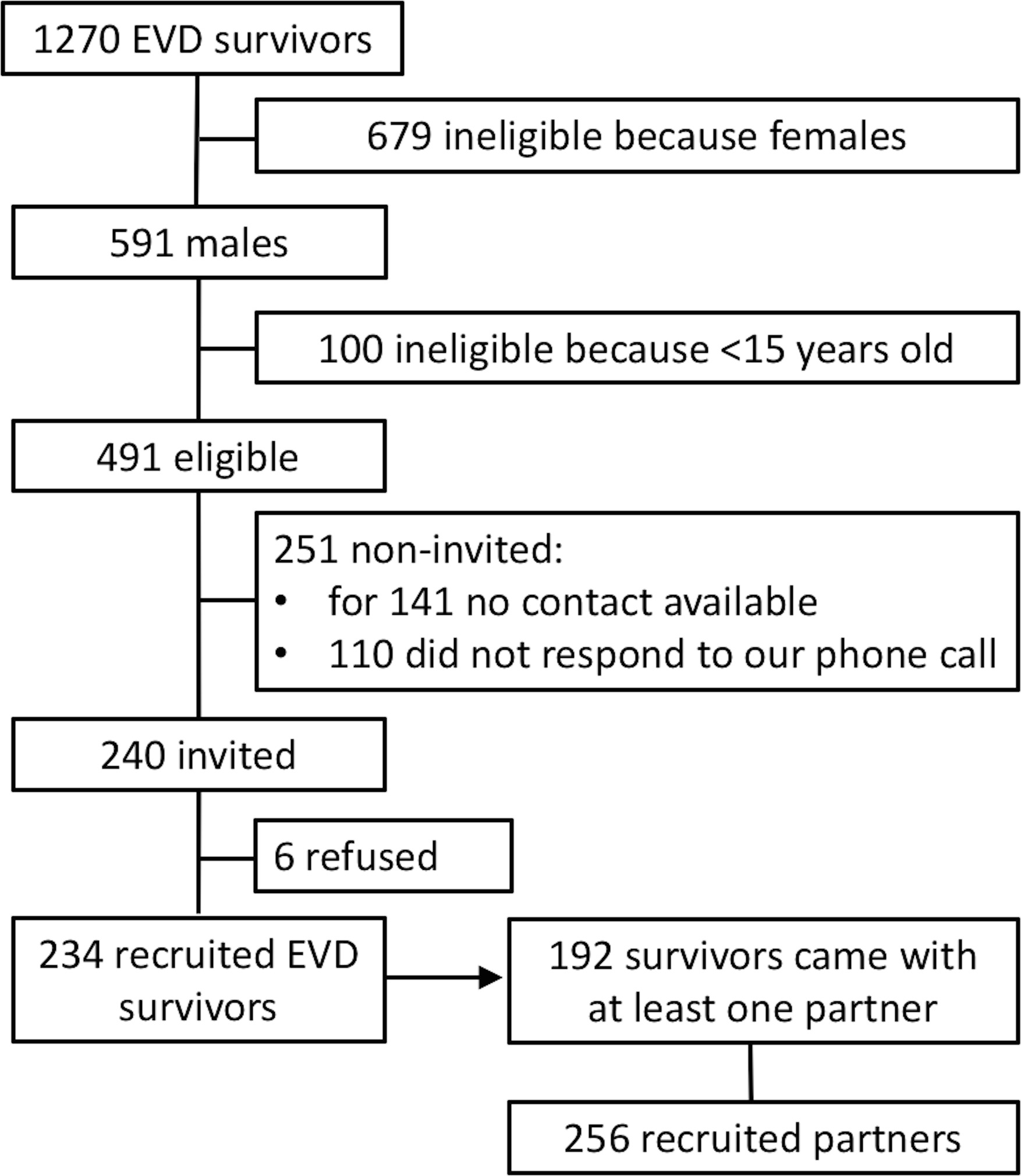

Supplement: Export to PDFSupplementary file 1 [file bmjgh-2017-000412supp001.jpg]
