# Supplementary material for: Sex practices and awareness of Ebola virus disease among male survivors and their partners in Guinea
Source: BMJ Glob Health. 2017 Sep 25;2(3):e000412. doi: 10.1136/bmjgh-2017-000412 (PMC5623339; doi:10.1136/bmjgh-2017-000412)
Supplement: Supplementary file 2 [file bmjgh-2017-000412supp002.pdf]

Supplementary Table 1: Risk factors for awareness of risk of Ebola virus sexual transmission amongst subgroups (1. EVD Survivors; 2. Sexual partners of survivors). n: participants with outcome (i.e. aware of the risk of Ebola virus sexual transmission, or score equal or higher than 4); N: total number of exposed individuals. cOR: crude odds ratio aOR: adjusted odds ratio.

	**1.EVD Survivors**			**2. Partners**		
	**n/N (%)**	**cOR (95% CI)**	**aOR* (95% CI)**	**n/N (%)**	**cOR (95% CI)**	**aOR* (95% CI)**
**All**	131/231 (56.7)			94/256 (36.7)		
**Reported having been informed by the partner**						
no	/	/	/	75/115 (65.2)	-	-
yes	/	/	/	12/112 (10.7)	15.6 (7.70-31.8)	20.5 (8.92-47.4)
**Age (years)**						
15-24	36/58 (62.1)	-	-	33/117 (28.2)	-	-
25-39	67/107 (59.3)	0.95 (0.50-1.83)	0.63 (0.29-1.37)	55/123 (44.7)	2.06 (1.20-3.52)	2.77 (1.28-5.98)
40-59	25/57 (43.8)	0.48 (0.23-1.01)	0.41 (0.17-1.02)	6/14 (42.9)	1.91 (0.62-5.92)	1.25 (0.26-5.87)
>60	3/9 (33.3)	0.31 (0.96-2.78)	0.32 (0.06-1.64)	0/0 (0)	-	-
**Zone of residence**						
Urban	72/110 (61.0)	-	-	48/130 (36.9)	-	-
Rural	59/113 (52.2)	0.72 (0.43-1.20)	0.64 (0.28-1.45)	46/126 (36.5)	0.98 (0.59-1.63)	0.59 (0.20-1.73)
**Years in Education**						
none	39/81 (48.1)	-	-	55/143 (38.5)	-	-
1-5	14/38 (36.8)	0.62 (0.28-1.35)	0.50 (0.21-1.19)	11/39 (28.2)	0.63 (0.29-1.36)	0.52 (0.17-1.50)
6-12	42/71 (59.1)	1.60 (0.84-3.03)	1.63 (0.70-3.77)	16/58 (27.6)	0.61 (0.31-1.19)	0.56 (0.16-1.92)
>13	35/40 (87.5)	6.43 (2.44-16.9)	10.1 (2.68-38.2)	11/15 (73.3)	4.40 (1.33-14.5)	2.54 (0.23-28.6)
**Employment**						
Housewife/Farmer/Labourer	39/94 (41.5)	-	-	45/134 (33.6)	-	-
State employee/Business owner	41/61 (67.2)	2.80 (1.44-5.46)	2.59 (1.19-5.61)	30/77 (39.0)	1.26 (0.68-2.76)	2.35 (0.97-5.66)
Other	49/74 (66.2)	2.71 (1.45-5.07)	0.95 (0.40-2.29)	18/44 (40.9)	1.37 (0.71-2.26)	1.88 (0.39-9.04)
**Region of residence**						
Conakry	37/68 (54.4)	-	-	31/85 (36.5)	-	-
Lower Guinea	41/70 (58.6)	1.26 (0.65-2.46)	3.06 (1.20-7.82)	17/80 (21.2)	0.47 (0.23-0.94)	0.58 (0.18-1.84)
Forested Guinea	53/93 (57.0)	1.15 (0.62-2.15)	4.51 (1.66-12.2)	46/91 (50.6)	1.78 (0.97-3.26)	1.51 (0.43-5.31)

*Adjusted for all other variables in the table, / : for survivors: 'Reported having been informed by the partner' is not part of the model.

Supplementary Table 2: Numbers of aware EVD survivors by region of residence and years in education. ssOR are not reported and no test for interaction was done because of data sparsity.
N: EVD survivors with the exposure status (region of residence) in corresponding 'years in education' stratum.

Years in education	Region of residence	% (aware survivors/N)	p-value*
None	Conakry	55 (6/11)	0.085
	Lower Guinea	29 (7/24)	-
	Forested Guinea	57 (26/46)	-
1-5 years	Conakry	40 (4/10)	0.96
	Lower Guinea	33 (3/9)	-
	Forested Guinea	37 (7/19)	-
5-12 years	Conakry	30 (7/23)	0.002
	Lower Guinea	80 (20/25)	-
	Forested Guinea	65 (15/23)	-
>13 years	Conakry	83 (20/24)	0.80 (Fisher's exact test)
	Lower Guinea	92 (11/12)	-
	Forested Guinea	100 (4/4)	-

*Chi^2 test unless specified

Supplementary Panel: Questions to assess awareness of EVD sexual transmission (≥4 points: aware)

a) Can Ebola persist in an individual after recovery? (1 point for "yes")

b) Can an individual shed Ebola after recovery? (1 point for "yes")

c) Which mode of transmission is the most likely after recovery? (multiple choice, 1 point for "sexual transmission")

d) In which body fluid can Ebola virus persist? (multiple choice, 1 point for semen, 0.5 points vagina, breast milk, eye, cerebrospinal fluid; maximum 2 points)

e) How long can the virus persist? (multiple choice, 1 point for "9 months or more")

Supplementary Figure: Flowchart for the recruitment of EVD survivors.
